# Effect of ropivacaine, mepivacaine or the combination of ropivacaine and mepivacaine for epidural anaesthesia on the postoperative recovery in patients undergoing caesarean section: a randomized, prospective, double-blind study

**DOI:** 10.1186/s12871-024-02413-z

**Published:** 2024-02-07

**Authors:** Muye Wang, Chen Liao, Xiaocui Li, Weiming Chen, Yujie Li, Wei Zhang, Shouping Wang

**Affiliations:** 1 Department of Anesthesiology, Guangdong Provincial Key Laboratory of Major Obstetric Diseases, Guangdong Provincial Clinical Research Center for Obstetrics and Gynecology, The Third Affiliated Hospital of Guangzhou Medical University, Guangzhou, China; 2 Department of Obstetrics and Gynecology, Center for Reproductive Medicine, Guangdong Provincial Key Laboratory of Major Obstetric Diseases, Guangdong Provincial Clinical Research Center for Obstetrics and Gynecology, Guangdong-Hong Kong-Macao Greater Bay Area Higher Education Joint Laboratory of Maternal-Fetal Medicine, The Third Affiliated Hospital of Guangzhou Medical University, Guangzhou, China; 3 Key Laboratory for Reproductive Medicine of Guangdong Province, The Third Affiliated Hospital of Guangzhou Medical University, Guangzhou, China

**Keywords:** Ropivacaine, Mepivacaine, Epidural anesthesia, Cesarean section

## Abstract

**Background:**

Anaesthetic methods and drugs with rapid onset, rapid recovery and better postoperative analgesia are more suitable for rapid recovery in obstetric anaesthesia. We formulated the following hypothesis: a combination of mepivacaine and ropivacaine could provide a longer analgesic effect and have more advantages in terms of rapid-recovery indicators.

**Methods:**

A total of 180 pregnant women scheduled to undergo elective caesarean sections were randomly assigned to three surgical groups, which received 2% mepivacaine (Group M), 2% mepivacaine + 0.75% ropivacaine (Group MR) (Volume 1:1) or 0.75% ropivacaine (Group R) through an epidural catheter. The situation of postoperative analgesia and other indicators of rapid recovery were recorded.

**Results:**

One hundred and fifty patients were included in the final analysis. Their demographic data were similar. The visual analogue scale (VAS) scores of Group MR and Group R were lower than Group M at 1 and 2 h after surgery both at rest and with movement (*P* < 0.05), and the time to first ambulation in Group MR (17.38 ± 2.06 h) and Group M (17.20 ± 2.09 h) was shorter than that in Group R (22.18 ± 1.74 h) (*P* < 0.05).

**Conclusion:**

Application of 2% mepivacaine combined with 0.75% ropivacaine for epidural anaesthesia can provide longer postoperative analgesia and earlier ambulation, these effect may be more suitable than that of 2% mepivacaine or 0.75% ropivacaine alone for caesarean section.

**Trial registration:**

This study was registered at Chinese Clinical Trial Registry (Registration number: ChiCTR 2300078288; date of registration: 04/12/2023).

## Background

Caesarean section is one of the most common surgical operations in the world, and it can also be a life-saving intervention when medically indicated [1, 2]. Adjustment of the sensory block level during surgery and good effects of muscle and postanaesthesia analgesia are provided in epidural anaesthesia with a catheter, demonstrating that epidural anaesthesia is the better choice for caesarean section [3]. The concept of enhanced recovery after surgery (ERAS) originated in the field of gastrointestinal surgery and has been gradually extended to the field of obstetric surgery [4]. With the improvement in operation methods and the shortening of operation time, anaesthesia with rapid onset of block, a short motor block duration to enable early exercise and a long sensory block duration to provide good analgesia after surgery has become the goal of caesarean section operation [5]. This is particularly important when anaesthetics that have long-lasting characteristics are used for epidural anaesthesia. Based on the above description, epidural anaesthesia combined with different local anaesthetics during caesarean sections is not rare [6].

In recent decades, ropivacaine has become popular for obstetric anaesthesia because of its dissociation of sensory and motor blocks. Several randomised double-blind studies have shown that epidural ropivacaine provides effectiveanesthesia without adversely affecting neonatal outcomes [7–10]. A study for elderly patients undergoing hemiarthroplasty demonstrated that epidural 0.75% ropivacaine with fentanyl provides more satisfying intraoperative hemodynamic fluctuations [11]. In a study involving 108 pregnant women, the comparison between epidural application of 0.75% ropivacaine (15 ml) and subarachnoid application of 0.75% ropivacaine (1.5 mg) for elective cesareansection revealed no significant differences in outcomes related to postoperative pain, analgesic requirements, patient satisfaction and adverse effects [12]. However, ropivacaine cannot provide a faster onset for epidural anaesthesia, similar to the speed of sensory block of bupivacaine [13–16]. In lumbar epidural anesthesia, comparative data suggests that 0.75% ropivacaine provide the same sensory and motor block as bupivacaine 0.5% [16]. It has also been reported that the relative anesthetic potency of 0.5% bupivacaine is approximately equivalent to that of 2% lidocaine [17]. As a local anaesthetic agent with intermediate potency and duration, the chemical structure of mepivacaine is similar to that of lidocaine, and its maximum dose is 1.3–2 times greater than that of lidocaine [18]. So we hypothesized that the potency of 2% mepivacaine is similar to 0.75% ropivacaine and it has been used successfully for caesarean section without adverse neonatal effects [19]. Furthermore, in a randomized double-blind study involving 60 patients undergoing carotid endarterectomy with cervical plexus block, the effect of anesthesia was similar in patients receiving 0.75% ropivacaine or 2% mepivacaine [20]. However, we did not find any studies that used ropivacaine and mepivacaine in combination for epidural anaesthesia.

We hypothesized that administering a combination of ropivacaine and mepivacaine through the epidural space in caesarean surgeries would provide a longer duration of postoperative analgesia with a shorter motor block to enhance recovery after surgery. The primary outcomes included VAS scores and the time to first ambulation in the three groups after surgery. The other indicators regarding recovery after surgery were second outcomes. Therefore, our study aimed to compare the effect of different local anaesthetics used in epidural surgery for caesarean section and their influence on recovery after surgery to provide a clinical reference.

## Methods

The present study was a prospective, double-blind, randomized trial approved by the institutional review board of the Third Affiliated Hospital of Guangzhou Medical University (registration number 202301239), and this trial was retrospectively registered at the Chinese Clinical Trial Registry (ChiCTR 2300078288), conducted in accordance with the Declaration of Helsinki.

After signing an informed consent form, a total of 180 pregnant participants who underwent elective caesarean surgeries under regional anaesthesia were enrolled from February 2023 to October 2023. The inclusion criteria were as follows: age 20–40 years; primipara; weight 58–84 kg; height 155–165 cm; 37 weeks or more of gestation for a single live foetus; American Society of Anesthesiologists (ASA) physical status II; and no other systematic disease. The exclusion criteria were contraindication for regional anaesthesia; foetal distress or abnormalities; cardiovascular or cerebrovascular disease; failure of puncture requiring general anaesthesia.

Participants were randomly allocated to receive epidural anaesthesia with 0.75% ropivacaine (Group R), epidural anaesthesia with 0.75% ropivacaine + 2% mepivacaine (volume 1:1) (Group MR) or epidural anaesthesia with 2% mepivacaine (Group M) using a computer-generated randomization method. Group allocation numbers were kept in sealed, opaque envelopes with sequential numbers, which were opened to assign participants to one of the three treatment groups after informed consent was obtained. The enrolling anesthesiologist was aware of the group assignment, but this information was not disclosed to the treating anesthesiologist. The treating anesthesiologist, who had a professional qualification certificate for more than 3 years, was not the same person as the enrolling anesthesiologist. An independent nurse, not involved in any part of the study and under the guidance of the enrolling researcher, prepared the drugs. The situation during surgery was recorded by the treating anesthesiologist, and outcomes after surgery were recorded by the anesthesia assistant. Both personnel were blinded to group allocation. Sixty participants were included in each group. The participants and outcome assessors were blinded to the group allocations.

Participants fasted and did not receive preanaesthetic medicine. After entering the operating room, each patient received a 500 ml infusion of lactated Ringer’s solution in advance, continuous routine monitoring, including electrocardiogram, non-invasive blood pressure and pulse oximetry, assessed every 3 min. Oxygen (2 L/min) was inhaled through a nasal catheter. The patient was placed in the lateral decubitus position, epidural puncture with Tuohy needle (17G) at L2-3 intervertebral space, epidural catheter inserted in the same manner. After negative aspiration for blood or CSF (cerebral spinal fluid), 3 ml of lidocaine was injected epidurally as a test dose. Five minutes later, in the absence of accidental intrathecal block, a total of 10–17 ml of 0.75% ropivacaine (H20140763) was injected through the epidural catheter in Group R, and 10–17 ml of 2% mepivacaine (H20110062) was injected in Group M. The same volume of the combination of 0.75% ropivacaine + 2% mepivacaine was given in Group MR, and the volume of solution was given according to the patient’s height. After the block, all of the participants were placed in the supine position with the left uterus displaced 15° by placement of a wedge. In addition, for patients who can still feel the cold feeling of placing ice on the skin below the T6 level, emergency injection of 3–5 ml solution is given through an epidural catheter 5 min before operation.and whose VAS score was > 2 points during the surgery (VAS: visual analogue scale, draw a 10 cm horizontal line on the paper, one end of the horizontal line is 0, indicating no pain; The other end is 10, indicating severe pain; The middle part indicates different levels of pain. Ask the patient to mark the level of pain on a horizontal line according to how they feel). If the total volume of the solution was higher than 20 ml, patients still complained about pain during the surgery; we viewed this as a failure of intrathecal anaesthesia requiring conversion to general anaesthesia and excluded those patients from the study. Immediately after suturing of the peritoneum, all patients received a dose of 1 ug/kg sufentanil diluted to 50 ml with normal saline by pump at a speed of 2 ml/h intravenous (i.v.) for 24 h [21]. A vasoconstrictor was administered i.v. when the systolic blood pressure decreased to 90 mmHg or by 30% from baseline values. Atropine was administered i.v. when the heart rate decreased to 50 beats/min. After surgery, a diclofenac sodium suppository was administered through the anus for rescue analgesia only when the patients complained of pain (VAS > 3). All outcomes were observed by anaesthesiologists blinded to the patient group and dose assignments. The following variations were recorded: 1) Latency of sensory block: time elapsed between the end of anesthetic epidural injecion to the absence of cold at T10 level; 2) Maximum sensory nerve block level: assessment of the sensory block level after injection of the anaesthetic solution until the level stabilized; 3) Total dose of anaesthetic medicine; 4) Neonatal outcomes: the 1st and 5th minute neonate Apgar score and the pH values of the neonatal umbilical vein blood gas were registered; 5) The quality of analgesia during the operation was divided into 4 grades according to the method developed by Lee et al. [22]: Excellent: no complaint of discomfort; Good: slight discomfort, no need for additional medication; Fair: uncomfortable, but controllable with intravenous benzodiazepines and/or opioids; poor: intravenous administration is uncontrollable and requires general anesthesia; 6) VAS score: pain assessments were performed at 0, 0.5, 1, 2, 4, 6, 8, and 12 h after leaving the operating room using a VAS score of 0–10, and the patients who needed rescue analgesia were recorded. 7) Modified bromage score: Modified bromage scores were assessed at 0,0.5,1,2,4,6,8, and 12 h after surgery according to the modified Bromage scale (MBS): 0 = free movement of the lower limbs (null), 1 = ability to flex the knees and move the feet, 2 = ability to only flex the feet, 3 = 0 = free movement of the lower limbs (null), 1 = ability to flex the knees and move the feet, 2 = ability to only flex the feet, 3 = complete inability to move the lower limbs. 8) Maternal haemodynamic variables: Mean arterial pressure (MAP, mHg), heart rate (HR, bpm), and pulse oximetry were evaluated every 5 min during surgery. 9) Post operative outcomes were recorded: full motor block recovery: time from the end of epidural anesthetic injection to free lower limb movement;ambulation: time from the end of surgery to first ambulation; supplemental analgesia until 12 h: the number of patient need supplemental analgesia until 12 h after surgery; gas removal: time from the end of surgery to first gas removal; urinary catheter removal: time from the end of surgery to urinary catheter removal; time to discharge: time from the end of surgery to discharge. General information of the patients, such as age, weight, height, gestational weeks, gravida, para, length of operation and blood loss volume, was also recorded. The primary outcomes, which were related to postoperative recovery, included the situation of postoperative analgesia and recovery function included VAS scores and the time to first ambulation after surgery. The indicators regarding to rapid recovery such as other postoperative outcomes were second outcomes.

### Statistical analysis

This was a randomized prospective trial comparing the anaesthetic effect and recovery between different anaesthesia medicines. The sample size estimation was based on the following data referenced by a literature search and application to parturients [23] in which the recovery index, such as the time to first mobilization, was 13.8 ± 2.9 h during ropivacaine use in epidural anaesthesia. We assumed that a difference of 2 h in time to first mobilization among the groups was clinically meaningful, with α = 0.05 and β = 0.1 (two-sided); the dropout rate was 5%, and the sample size was 50 people in each group.

Data are presented herein as means and standard deviations or medians (interquartile distances) for continuous variables and as frequencies and proportions for categorical variables. Continuous data were compared among the three groups using one-way ANOVA tests for normally distributed variables and Kruskal‒Wallis rank sum tests for nonnormally distributed variables. For rank data, the Kruskal‒Wallis rank sum test was also used for comparison. Count data were analysed using χ2 tests or Fisher’s tests. Analyses were performed using SPSS 26.0 (IBM, Armonk, NY, USA). Statistical significance was defined as *P* < 0.05 (two-sided).

## Results

**Fig. 1:**
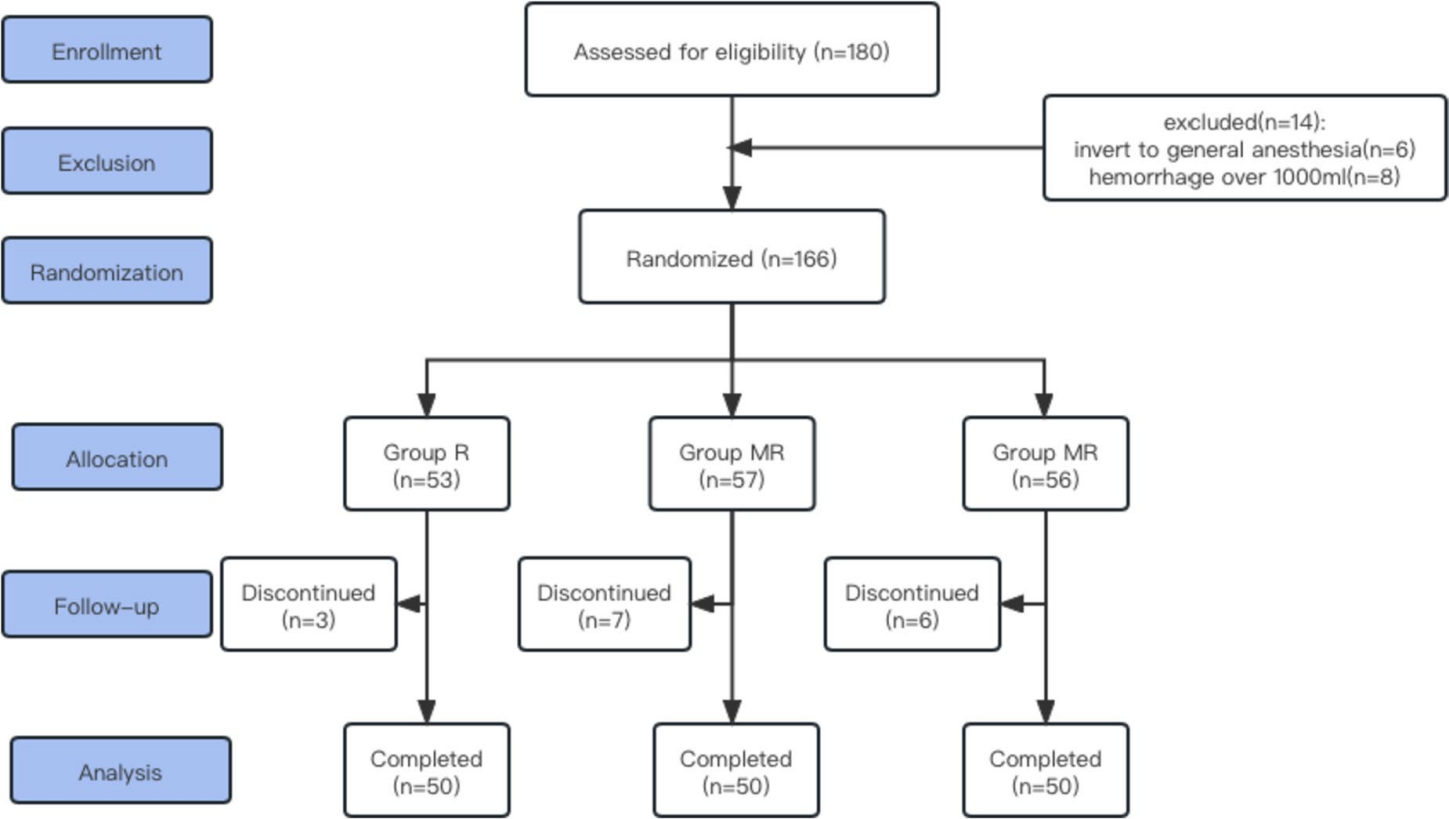
Flow diagram of patient inclusion procedure. Of 180 patients selected for eligibility initially, fourteen were excluded. The leaving 166 were randomly allocated to three groups to receive epidural anaesthesia with 0.75% ropivacaine (Group R), epidural anaesthesia with 0.75% ropivacaine + 2% mepivacaine (Group MR) or epidural anaesthesia with 2% mepivacaine (Group M). Sixteen patients discontinued the study after group assignment, with 150 patients remaining for the final analysis

A total of 180 pregnant women met the inclusion criteria and agreed to participate. Six patients withdrew from the study because of the failure of epidural anaesthesia (*n* = 6), loss of data collection (*n* = 16), and haemorrhage over 1000 ml (*n* = 8). A total of 150 parturients (50 in Group R, 50 in Group MR and 50 in Group M) completed the study (Fig. 1). There was no significant difference in demographic characteristics (age, weight, height, gestational week), and there was no significant difference in operation time, haemorrhage or foetus extraction time, thus reducing the bias that may be related to the operation. A significant difference was not detected among the groups in the incidence of adverse reactions (Table 1).

**Table 1:** Characteristics, surgery data and adverse reactions according to the groups

	Group R(*n* = 50)	Group MR(*n* = 50)	Group M(*n* = 50)	*P* value
**Characteristics**				
Age (years)	29.82 ± 3.91	28.80 ± 3.53	28.48 ± 4.04	0.193
Height (cm)	159.48 ± 3.64	159.41 ± 3.71	159.74 ± 3.91	0.910
Weight (kg)	68.15 ± 5.39	68.42 ± 5.48	68.10 ± 5.38	0.951
Gestational (weeks)	38.08 ± 1.00	38.18 ± 0.92	38.12 ± 0.97	0.863
**Surgery**				
Haemorrhage (ml)	[300(300,400)]	[300(300,400)]	[300(300,362.5)]	0.857
Duration of surgery (min)	68.16 ± 8.98	68.12 ± 7.42	67.52 ± 7.78	0.899
Time for foetal extract (min)	19.42 ± 3.13	19.48 ± 3.06	19.62 ± 3.23	0.948
**Adverse reactions**				
Nausea and vomit	5(10%)	7(14%)	6(12%)	0.993
Bradycardia	0	0	0	
Nervous system symptoms	0	0	0	

Data are presented as mean score ± SD or interquartile range [IQR (range)] or n (%) of patients. Data were analyzed using analysis of variance (continuous variables) or the χ^2^ test (incidence variables), Kruskal–Wallis rank sum test was also used for rank data. Group R: epidural anaesthesia with 0.75% ropivacaine; Group MR: epidural anaesthesia with 0.75% ropivacaine + 2% mepivacaine (Volume 1:1); Group M: epidural anaesthesia with 2% mepivacaine; *SD* standard deviation; *IQR*: interquartile range;There were no significant differences among the groups (*P* > 0.05)

### Block characters

The time needed for the sensory block to reach the T10 level was similar among the three groups (*P* > 0.05), while the time needed for the sensory block to reach its maximum level was similar in Groups M and MR and shorter than that in Group R (*P* < 0.001). The maximum block level was similar in all groups (*P* > 0.05). The total amount of local anaesthetics used to obtain a sufficient sensory block level to start the operation was 18.15 ± 1.59 ml in Group R, 18.02 ± 1.76 ml in Group MR and 18.12 ± 1.67 ml in Group M (*P* > 0.05) (Table 2).

**Table 2:** The situation of sensory and motor block induced by drugs and the doses of drugs

	Group R (*n* = 50)	Group MR (*n* = 50)	Group M (*n * = 50)	*P* value
Time for sensory block to reach T10 (min)	9.38 ± 1.67	9.48 ± 1.58	9.06 ± 1.62	0.403
Time for sensory block to reach its maximum (min)	19.26 ± 1.67^a^	16.62 ± 1.63	16.12 ± 1.7	< 0.001
Maximum sensory block level	T6 (T6,T6)	T6 (T6,T6)	T6 (T6,T6)	0.366
Doses of drugs (ml)	18.15 ± 1.59	18.02 ± 1.76	18.12 ± 1.67	0.921

Data are presented as mean score ± SD or interquartile range [IQR (range)]. Data were analyzed using analysis of variance (continuous variables) or the Kruskal–Wallis rank sum test for rank data. Group R: epidural anaesthesia with 0.75% ropivacaine; Group MR: epidural anaesthesia with 0.75% ropivacaine + 2% mepivacaine (Volume 1:1); Group M: epidural anaesthesia with 2% mepivacaine

*SD* standard deviation, *IQR* interquartile range

^a^The time for sensory block to reach its maximum: There were significant differences between Group R and Group MR as well as between Group R and Group M (*P* < 0.001) (The *p*-values are based on Scheffe’s post-hoc test adjusted using the Bonferroni correction), and there was no significant difference between Group M and Group MR (*P* > 0.05)

### Analgesic quality

The intra-operative analgesic effects were rated as “excellent”: 44 (88%), 46 (92%), and 44 (88%), respectively. There was no difference about the satisfactory quality of analgesia between the three groups. (*P* > 0.05) (Table 3).

**Table 3:** Quality of analgesia

	Group R (*n* = 50)	Group MR (*n* = 50)	Group M (*n* = 50)	*P* value
Excellent	44 (88%)	46 (92%)	44(88%)	0.844
Good	3 (6%)	3 (6%)	4 (8%)	1.000
Fair	3 (6%)	1 (2%)	2 (4%)	0.871
Bad	0 (0%)	0 (0%)	0 (0%)	

Data are presented as n (%) of patients. Data were analyzed using analysis of the χ2 test (incidence variables). Group R: epidural anaesthesia with 0.75% ropivacaine; Group MR: epidural anaesthesia with 0.75% ropivacaine + 2% mepivacaine (Volume 1:1); Group M: epidural anaesthesia with 2% mepivacaine; There were no significant differences among the groups (*P* > 0.05)

### VAS  and MBS scores

**Fig. 2:**
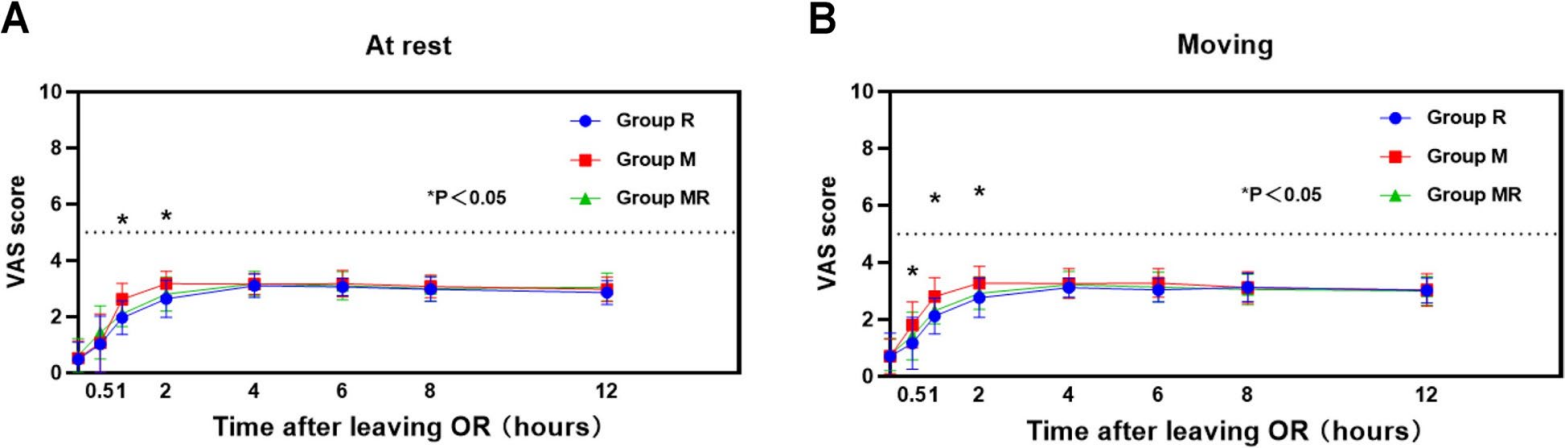
Comparison of the analgesic effects of epidural anaesthesia after surgery. Group R: epidural anaesthesia with 0.75% ropivacaine; Group MR: epidural anaesthesia with 0.75% ropivacaine + 2% mepivacaine (Volume 1:1); Group M: epidural anaesthesia with 2% mepivacaine; OR: operating room; VAS score: 0–10 visual analogue scales. There was no significant difference between Group R and Group MR in the VAS scores at rest or with movement; however, the VAS scores at rest in Group M in 1 and 2 h after leaving the OR were higher than those in Group R or Group MR. The VAS scores at the timepoints of 0.5, 1, and 2 h after leaving the OR in Group M were also higher than those in the other two groups when moving. In addition, there was no significant difference in VAS score among the three groups at 0.5 h after leaving the OR at rest and 0,4, 6, 8, and 12 h after leaving the OR, whether at rest or moving

VAS score comparison showed that VAS at rest is as follows: VAS at rest in Group M was higher than Group MR and Group R at 1,2 h after leaving the OR (operate room)(*P* < 0.05), while there were no significant difference between Group MR and Group R (*P* > 0.05), VAS in moving in Group M was higher than Group MR and Group R at hours 0.5,1,2 after leaving the OR (*P* < 0.05), while there were no significant difference between Group MR and Group R (*P* > 0.05). There was no significant difference in VAS score among the three groups at 0.5 h after leaving the OR at rest and 0,4, 6, 8, and 12 h after leaving the OR (*P* > 0.05), regardless of whether they were in rest or moving (Fig. 2).

**Fig. 3:**
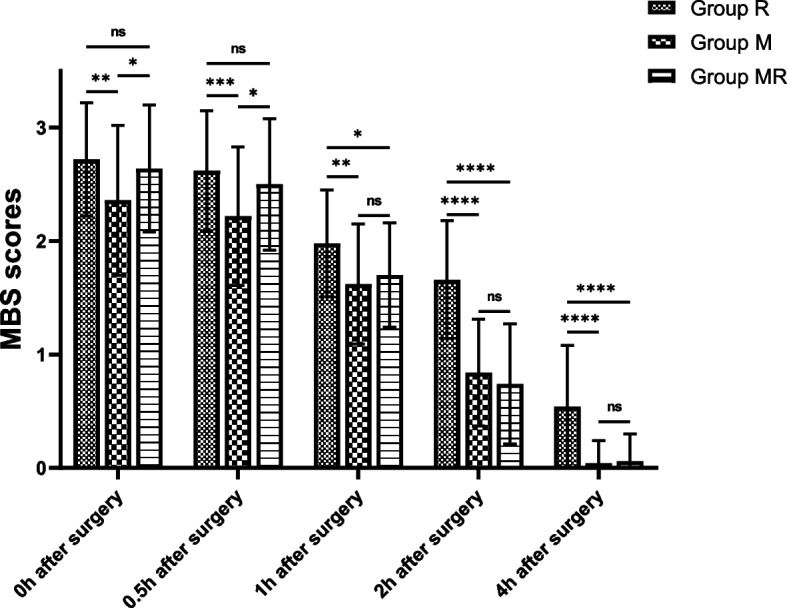
Comparison of the MBS scores for epidural anaesthesia. Group R: epidural anaesthesia with 0.75% ropivacaine; Group MR: epidural anaesthesia with 0.75% ropivacaine + 2% mepivacaine (Volume 1:1); Group M: epidural anaesthesia with 2% mepivacaine; The MBS scores in Group R or Group MR were higher than those in Group M, while there was no significant difference between Group R and Group MR immediately after surgery. At 0.5 h after surgery, the MBS scores were higher in Group R than in Group M, while there were no difference comparing Group R with Group MR or Group M with Group MR. At 1 h, 2 h or 4 h after surgery, the MBS scores were higher in Group R than in Group M or Group MR, and there were no significant differences between Group M and Group MR

The MBS scores in Group R and Group MR were higher than those in Group M (*P* < 0.05), while there were no significant differences between Group R and Group MR (*P* > 0.05) immediately after surgery. At 0.5 h after surgery, the MBS score was higher in Group R than in Group M (*P* < 0.05), while there were no differences between Group R and Group MR or between Group M and Group MR (*P* > 0.05). However, at 1 h, 2 h or 4 h after surgery, the MBS score was higher in Group R than in Group M or Group MR (*P* < 0.05), and there were no significant differences between Group M and Group MR (*P* > 0.05); in particular, the MBS scores were 0.06 ± 0.24, 0.04 ± 0.20 in Group M and Group MR at 4 h after surgery, the majority of patients have regained their lower limb strength (Fig. 3).

### Infant information and postoperative outcomes

The pH value of the umbilical cord venous blood and the Apgar score at 1 and 5 min of neonates after delivery were in the normal range. There was no significant difference in any of the indices among the three groups (all *P* > 0.05). The complete recovery time of motor block and the time to ambulation were 3.07 ± 0.77 h and 22.18 ± 1.74 h, respectively, which were significantly longer (*P* < 0.05) for the patients receiving ropivacaine epidurally (Group R) than for the participants epidurally receiving the combination of mepivacaine and ropivacaine (Group MR) or mepivacaine only (Group M). The incidence of supplemental analgesia in three groups shows no significant difference until 12 h after surgery (*P* > 0.05). The time of first gas removal was earlier in Groups M and MR than in Group R (*P* < 0.05). Differences between the groups with respect to time to urinary catheter removal or time to discharge were not detected (all *P* > 0.05) (Table 4).

**Table 4:** Infant information and postoperative outcomes

	Group R (*n * = 50)	Group MR (*n* = 50)	Group M (*n* = 50)	*P* value
**Infant information**				
Apgar score at 1 min	9.94 ± 0.24	9.96 ± 0.20	9.92 ± 0.34	0.755
Apgar score at 5 min	9.98 ± 0.14	10.00 ± 0.00	9.96 ± 0.20	0.365
pH	7.35 ± 0.25	7.35 ± 0.24	7.35 ± 0.25	0.978
**Postoperative outcomes**				
Full motor block recovery (h)	3.07 ± 0.77^b^	1.60 ± 0.52	1.55 ± 0.44	< 0.001
Ambulation (h)	22.18 ± 1.74^c^	17.38 ± 2.06	17.20 ± 2.09	< 0.001
Supplemental analgesia until 12 h	9(18%)	11(22%)	13(26%)	0.665
Gas removal (h)	21.42 ± 2.48^d^	19.64 ± 2.92	19.92 ± 2.91	0.033
Urinary catheter removal (h)	21.47 ± 2.28	22.33 ± 1.69	21.92 ± 1.98	0.102
Time to discharge (h)	70.52 ± 3.28	70.02 ± 3.00	71.14 ± 2.96	0.657

Data are presented as mean score ± SD or n (%) of patients. Data were analyzed using analysis of variance (continuous variables) or the χ^2^ test (incidence variables). Group R: epidural anaesthesia with 0.75Group R: epidural anaesthesia with 0.75% ropivacaine; Group MR: epidural anaesthesia with 0.75% ropivacaine + 2% mepivacaine (Volume 1:1); Group M: epidural anaesthesia with 2% mepivacaine; *SD* standard deviation; the values are the number (proportion) or mean (SD) as appropriate. pH: neonatal umbilical vein blood gas

^b^Time to full motor block recovery: the time to full motor block recovery (MBS = 0) in Group R was longer than that in Group M or Group MR (*P* < 0.05), and there were no significant differences between Group M and Group MR (*P* > 0.05)

^c^Time to ambulation: the time to ambulation in Group R was longer than that in Group M or Group MR (*P* > 0.05), while there were no significant differences in Group M and Group MR (*P* < 0.05)

^d^Time to gas removal: the time to gas removal in Group R was longer than that in Group M or Group MR, while there were no significant differences between Group M and Group MR (*P* > 0.05). There were no significant differences in the infants’ Apgar score at 1.5 min, the pH value of neonatal umbilical vein blood gas, the rate of supplemental analgesia until 12 h after surgery, the time to urinary catheter removal, or the time to discharge among the three groups (*P* > 0.05) (The* P*-values are based on Scheffe’s post-hoc test adjusted using the Bonferroni correction)

## Discussion

In our study, three anaesthetic drugs were selected for the anaesthesia in caesarean section. The results showed that none of the mothers had adverse reactions, such as bradycardia or nervous system symptoms, but some of them experienced nausea and vomiting, which may have been caused by a traction due to insufficient anaesthetic levels or reaction by oxytocin medication. The 1st and 5th minute Apgar scores and the pH value of the umbilical venous blood of neonates were also within the normal range and had no side effects on the newborns. Furthermore, there was no significant difference in analgesic quality among the three groups or the incidence of supplemental analgesia until 12 h after surgery among the three groups, indicating that the three anaesthesia regimens in this study integrated well.

Our study showed that 0.75% ropivacaine combined with 2% mepivacaine epidural anaesthesia could shorten the time of sensory block to reach the maximum level compared with 0.75% ropivacaine alone, and the duration of anaesthesia was longer than that with 2% mepivacaine according to the VAS scores. The time to first ambulation in the 0.75% ropivacaine group was significantly longer than that in the 2% mepivacaine group and combination group.

The pH and pKa characteristics of local anaesthetics can affect the onset of action. In a study comparing standard 2% mepivacaine with 2% alkalized mepivacaine epidural for cesarean section, it was observed that alkalizedmepivacaine shortened the time to onset of action. Additionally, the total dose administered in the two groups showed no significant difference, with values of 21.3 ml and 18.9 ml, respectively [24]. Previous papers have shown that the pKa of ropivacaine is 8.05 and that of mepivacaine is 7.7 [20, 24]. As we observed, mepivacaine provided a faster onset of sensory block to its maximum level, perhaps because the pKa of mepivacaine is proximate to the tissue pH. When 1% mepivacaine was injected epidurally at different speeds, more dermatomes were blocked in the faster group 5 min later, while 16 dermatomes were blocked 15 min later; moreover, there was no significant difference between the fast group and the slow group, indicating that the anaesthetic effect could reach the peak after 15 min regardless of how fast or slow the injection speed was [25].Our research reveals that epidural application of mepivacaine can deliver effective anesthesia without causing adverse reactions in mothers and infants [24, 26, 27].

Previous literature has shown that the use of 20 ml 0.75% ropivacaine for epidural anaesthesia during caesarean section can provide the maximum level of sensory block to T6, which takes 23 min [28]. Similar results were obtained by Bjqrnestad et al. [16]. All of these studies confirm our findings on the time it takes for Group M and Group R to reach maximum sensory block.

The results showed that when using 20 mL 0.75% ropivacaine in the epidural space for elective caesarean section, the duration of motor block was 237 min [29], similar to our results; in Group R, the time to full motor block recovery was 3.07 ± 0.77 h. When mepivacaine was administered in the epidural space, it provided a slightly longer duration of action [4, 18]. Mepivacaine is also frequently combined with longer-acting local anaesthetics such as ropivacaine, providing a quick-onset anaesthetic that also has the benefits of a prolonged duration [30].

In previous studies, local anaesthetic combinations were used for regional anaesthesia, creating favourable results with regard to the onset and duration of anaesthesia [31–33]. The literature published by Cubillon et al. [34] compared mixtures of long-acting local anaesthetics such as ropivacaine or bupivacaine with lidocaine-induced faster onset blocks and decreased duration for femoral-sciatic nerve blockade. When a mixture of 1.5% mepivacaine (15 ml) and 0.5% bupivacaine (15 ml) was used to block the brachial plexus under ultrasound guidance, Gadsden J [35] found that the duration of anaesthesia in the mixed solution group was longer than that of 30 ml 1.5% mepivacaine alone and shorter than that of 30 ml 0.5% bupivacaine alone. Gilles Guerrier et al. [36] used 2% mepivacaine combined with 0.75% ropivacaine for regional anaesthesia in in vitro-retinal surgery. The results of these papers were similar to our research, which indicates that a faster sensory block onset and longer analgesia duration can be obtained by combining mepivacaine and ropivacaine.

The concept of rapid recovery originated in 1995, Bardarm et al. [37] found that when the perioperative management was changed, eight high-risk elderly colorectal surgery patients were discharged from the hospital only two days after surgery. Since the establishment of ERAS in 2010, a series of procedural guidelines on rapid rehabilitation have been issued, laying the foundation for rapid surgical rehabilitation [38]. Since then, the application of the concept of rapid rehabilitation in gastrointestinal surgery has achieved remarkable results, successfully reducing the occurrence of postoperative complications, shortening the length of hospital stay, and reducing the burden of hospital expenses for patients [39, 40]. Mothers need to recover as soon as possible to take care of themselves and their newborns. Therefore, the promotion of ERAS in caesarean section is of great significance [41]. Vanderbilt Medical Center emphasizes that the core components of rapid recovery from caesarean section surgery include promotion of early mobilization, prevention of nausea and vomiting, promotion of gastrointestinal function recovery, multimodal analgesia, early removal of the urinary catheter, prevention of venous thrombosis of the lower limbs, and early discharge from the hospital [42, 43]. In the study parallel to ours, the time of first mobilization and gas removal were shorter and the analgesia time was longer in the combined group, which was because the motor block was shorter than that in Group R and the sensory block was longer than that in Group M, which is a favourable result for patients undergoing caesarean section in epidural anaesthesia. In a study of 416 patients undergoing abdominal surgery, ambulation within a day of surgery shortened hospital stays, demonstrated the achievement of early postoperative mobilization is one of the important components for early recovery [44]. Similar findings were presented in a multicenter study of 23,295 patients in spine surgery, which indicated that ambulation on the day of surgery reduced the incidence of urinary retention, ileus and the length of stay [45]. In our study, we also found that all three groups walked around within one day after surgery, and the Group MR and the Group Meipvcaine walked earlier than the Group R alone. However, there was no significant difference in the time to urinary catheter removal and the time to discharge among the three groups, which may be due to the unified management of the time of catheter removal and patient discharge in the ward rather than individual management.

## Conclusion

In conclusion, our results indicate that a combination of 2% mepivacaine with 0.75% ropivacaine provides a shorter time to first ambulation compared with 0.75% ropivacaine alone, while provides longer analgesia and lower VAS scores until 2 h compared with 2% mepivacaine alone. In epidural anaesthesia, a combination of 0.75% ropivacaine-2% mepivacaine promote the recovery of patients undergoing caesarean section and may be more suitable than 2% mepivacaine or 0.75% ropivacaine alone.

## Data Availability

No datasets were generated or analysed during the current study.

## Abbreviations

VAS: Visual analogue scale
ASA: American Society of Anesthesiologists
MBS: Modified Bromage scale
MAP: Mean arterial pressure
HR: Heart rate
i.v.: Intravenous
CSF: Cerebral spinal fluid
SD: Standard deviation
IQR: Interquartile range
OR: Operate room
ERAS: Enhanced recovery after surgery
SB: Sensory block
